# Factors influencing the intention of women with perinatal depression and their spouses to seek professional psychological help: a cross-sectional latent profile analysis

**DOI:** 10.3389/fpubh.2025.1544463

**Published:** 2025-04-08

**Authors:** Qinhan Zou, Xianliang Liu, Yingzi Yang, Yuelai Yang, Xia Duan

**Affiliations:** ^1^Nursing Department, Shanghai First Maternity and Infant Hospital, School of Medicine, Tongji University, Shanghai, China; ^2^School of Nursing and Health Sciences, Hong Kong Metropolitan University, Homantin, Hong Kong SAR, China; ^3^Nursing Department, Huadong Sanatorium, Wuxi, China; ^4^Department of Anesthesiology, Shanghai Ninth People’s Hospital Affiliated to Shanghai Jiao Tong University School of Medicine, Shanghai, China

**Keywords:** perinatal depression, intention, professional psychological help, influencing factors, latent profile analysis, spouses

## Abstract

**Background:**

Seeking professional psychological help can improve outcomes for women with perinatal depression (PND). However, the use of professional psychological help-seeking behaviors for women with PND is not promising. Spouses are important sources of support and play a decision-making role in the process of seeking professional psychological help for women with PND. Exploring the factors that influence couples’ intention to seek professional psychological help is important for developing effective interventions. This study aims to investigate the level and latent profiles of intention to seek professional psychological help in women with PND and their spouses, and identify influencing factors associated with different profiles.

**Methods:**

A cross-sectional study involving 267 women with PND and 267 spouses was conducted. The General Help-Seeking Questionnaire-the Intention to Seek Professional Psychological Help (GHSQ-ISPH), the Questionnaire of Stigma for Seeking Professional Psychological Help Questionnaire, the Attitude Toward Seeking Professional Psychological Help Scale, the Self-Efficacy for Seeking Mental Health Care Help Scale and the Edinburgh Postnatal Depression Scale were used. Latent profile analysis was used to identify groups with similar levels of GHSQ-ISPH. Multiple logistic regression was used to explore influencing factors associated with the intention to seek professional psychological help.

**Results:**

The mean GHSQ-ISPH scores of women with PND and their spouses were 12.17 ± 4.06 and 12.61 ± 3.88, respectively. LPA yielded three profiles. The profiles were named based on the GHSQ-ISPH score to reflect the level to which women with PND and their spouses intend to seek professional psychological help: “Women low-Spouses high intention for help” (Profile 1), “Women high-Spouses low intention for help” (Profile 2) and “Couple high intention for help” (Profile 3). Multiple logistic regression showed that spouses with a monthly income (858-1285/USD), with lower openness to seeking treatment for emotional problems, lower knowledge competence beliefs, and negative attitudes toward seeking professional psychological help were significantly associated with Profile 2 than with Profile 3 (*P* < 0.05 or *P* < 0.001). PND women who received a university or college and with lower public stigma were more significantly related to Profile 1 than Profile 3 (*P* < 0.05).

**Conclusion:**

The findings of this study indicate a moderate level of intention to seek professional psychological help among women experiencing PND and their partners. These results offer valuable insights for perinatal nurses, suggesting the necessity for the development of targeted interventions aimed at enhancing couples’ intentions to pursue professional psychological support. Identifying the factors associated with each profile outlined in this research offers the opportunity for more personalized and effective interventions. Tailored approaches like these could substantially enhance engagement with mental health resources, leading to improved outcomes for couples affected during the perinatal period.

## 1 Introduction

Perinatal depression (PND) includes prenatal depression and postpartum depression (PPD), which is a depression that occur during a women’s pregnancy, after delivery, or after miscarriage (1, 2). The incidence of PND in Chinese women is 16.3%, which is significantly higher than in other countries (3, 4). Untreated PND can have profound impacts on the well-being of the women, their partners, and the social, emotional, and cognitive development of their babies, with the most serious outcome being suicidal behavior in women (5–7). With its high prevalence and serious consequences, PND has become a significant concern in the world.

Seeking professional psychological help is an effective way for individuals to seek help from health professionals (e.g., psychologists, psychiatrists, mental health professionals and perinatal nurses) to cope with PND (8). Women with PND who have sought professional psychological help have reported relief from depressive symptoms, more positive coping with PND, and improved relationships with their babies and other family members (9). Although seeking professional help can prevent and manage mental health problems and protect women with PND from suicide (10). Currently, the utilization of professional psychological for women with PND is not promising in many countries, particularly in China. In Australia, 23% of women have sought help from healthcare professionals for emotional health during pregnancy (11), and 12.7% of women have sought health services for their depression symptoms in African (12). However, in China, only 9.3% of women with PND express a willingness to seek professional psychological help (13).

Due to the high incidence of PND and the low proportion of women with PND seeking professional psychological help, the research on women seeking professional psychological help for PND has become a hot research topic. Previous research (1, 11, 14–16) have found that the inability to recognize symptoms of PND, time constraints, lack of childcare, transportation, after delivery, cost and “sitting the month”/ “doing the month” are the barriers for women with PND to seek professional psychological help, while support from social support, location of services and a good understanding of PND are facilitating factors. The phrases “sitting the month” or “doing the month” refer to a traditional practice among Chinese women that encompasses postpartum health care. This practice typically involves a month-long period during which new mothers remain at home to rest and recuperate following childbirth (17). Women typically seek support from their spouses when dealing with PND, and spouses play a significant role in encouraging them to seek professional psychological help (18, 19). Despite these findings, there has been inadequate focus on addressing the reluctance of women with PND to seek professional psychological assistance. Insufficient access to formal support services significantly intensifies the long-term economic burden associated with mental health care during the recovery process. Consequently, further research is critical to identify and analyze the determinants influencing the willingness of women experiencing PND and their partners to seek professional psychological assistance.

Currently, the majority of research addressing the factors that influence the willingness to seek professional psychological help has been conducted overseas (1, 14, 19), with a focus on women experiencing PND, while some studies have also targeted spouses (20, 21). Although some research (13, 22–24) has been carried out in China to explore women’s intention to seek professional psychological assistance, several influencing factors have been identified. These include stigma, attitudes toward mental health, traditional cultural beliefs, and knowledge and perceptions of PND. Additionally, specific groups, such as Zhuang women with lower family support and other ethnic minority women with salaried employment in rural areas of western China, have been found to be particularly affected by these factors. Particularly in traditional culture, most Chinese women believe that abnormal emotions can be managed by themselves, often leading to a preference for seeking help from family members especially spouses (23, 25). This cultural tendency may significantly influence the current trend among Chinese women, which is characterized by a low propensity to seek professional help and a delayed transition from intention to actual help-seeking behavior (23, 26). Recognizing the importance of spousal support in shaping help-seeking behavior, a qualitative study (27) conducted by our research team disclosed that a vague understanding of PND and a lack of trust in obstetric providers were barriers preventing spouses from supporting their wives with PND in seeking formal help. However, the findings of this study, like many qualitative investigations, are limited in their representativeness and generalizability. Furthermore, there is a lack of research examining the factors influencing both women’s and their spouses’ intention to seek psychological professional help for PND in China. This gap in the literature is concerning, given the critical role that family dynamics, particularly spousal involvement, play in decision-making processes within Chinese cultural contexts. This study goes beyond merely investigating the association between spousal support and women’s help-seeking intentions by analyzing the distinct factors affecting the intentions of both women and their spouses. To address this gap, this study is the first to explore the factors that influence the willingness of women with PND and their spouses to seek professional psychological help. By examining these factors, this research aims to provide a deeper understanding of the interplay between cultural norms, spousal support, and help-seeking behaviors. Furthermore, the findings will inform the development of culturally tailored health strategies that can positively influence couples’ decision-making and encourage timely access to professional psychological care.

Therefore, this study aimed to (1) explore the intention to seek professional help among women experiencing PND and their spouses (2), classify the levels of intention to seek professional help among women experiencing PND and their spouses, and identify the differences in each profile and their influencing factors. The findings of this study will provide crucial evidence for perinatal nurses to devise targeted intervention strategies and enhance couples’ intention to seek professional psychological assistance.

## 2 Methods

### 2.1 Study design and setting

This study was a cross-sectional study conducted in the outpatient clinics and wards of a tertiary hospital in Shanghai, China, from June to September 2023.

### 2.2 Participants

The study inclusion criteria for women with PND were (1) ≥18 years old; (2) the Edinburgh Postpartum Depression Scale (EPDS) score≧9 points; (3) able to read, write, and speak Chinese; (4) willing and committed to participate in this study. The study inclusion criteria for spouses who were (1) spouses of women with PND; (2) ≥18 years old; (3) able to read, write, and speak Chinese; (4) willing and commit to participate in this study. The exclusion criteria for women with PND and spouses was that they had serious mental or psychiatric disorders (e.g., schizophrenia and bipolar disorder).

The sampling method was convenience sampling and the sample size was calculated according to the sample size calculation formula “*N* = Z^2^P(1-P)/d^2^” (*N*=Sample size, *Z* = Z statistic for a level of confidence, *P* = Expected prevalence, and *d* = Precision) (28). The prevalence of PND in Chinese women was 16.3% (3), with 1.96 for a 95% confidence level and *d* = 0.05. The calculations determined that a minimum sample size of 210 women with PND and 210 spouses was required.

### 2.3 Research team and data collection

The research team consisted of two experts and four nursing researchers. Prior to conducting formal surveys, researchers underwent standardized training to ensure the consistent interpretation of questionnaire responses. The research team was responsible for overseeing the quality of the study design and implementation process, ensuring that the research process was both scientifically sound and methodologically rigorous.

Potential participants were recruited through referrals by obstetricians, as well as outpatient and inpatient nurses, who then directed them to the researchers. The researchers screened participants who met the study inclusion and exclusion criteria. Participants were given detailed information and explanations and asked to sign an informed consent form. We conducted the survey using hard-copy questionnaires. The questionnaire design included detailed instructions to ensure participants fully understood the questions. The research investigators accompanied the participants in completing the questionnaire and observed whether the participants had read the questionnaire carefully to ensure the authenticity of the data. After completing the questionnaires, the research investigators checked each for completeness and accuracy.

### 2.4 Outcome variables and methods of assessment

#### 2.4.1 Socio-demographic information

Socio-demographic questionnaires were developed based on literature (29–31) and expert consultation. The socio-demographic questionnaires for women with PND and their spouses included demographic data (such as age, education, marital relationship), obstetrical information (such as stage of pregnancy, number of children) and mental health information (such as history of seeking psychological help).

#### 2.4.2 Edinburgh postnatal depression scale (EPDS)

Edinburgh Postnatal Depression Scale (EPDS), a 10-item questionnaire developed by Cox et al. (32) in 1987, is currently used to measure depression in women during the perinatal period (33). It was translated into Chinese by Lee et al. (34) in 1988. The scale used a 4-point Likert scale ranging from 0 (never) to 3 (often). The total score ranged from 0 to 30. A score of 9–12 indicated mild depression, and a score of 13–30 indicated severe depression. The Cronbach’s alpha coefficient of the Chinese version of EPDS was 0.82, indicating that it can be used to screen women with depression during the perinatal period (22). The Cronbach’s alpha coefficient for this study was 0.912.

#### 2.4.3 Attitudes toward seeking professional psychological help scale–short form (ATSPPH-SF)

The Chinese version of the Attitudes Toward Seeking Professional Psychological Help Scale–Short Form (ATSPPH-SF) is a 10-item scale translated by Hao et al. (35) to measure an individual’s attitude towards seeking professional psychological help (33). ATSPPH-SF consisted of two dimensions: openness to seeking treatment for emotional problems and value and need in seeking treatment. The scale used a 4-point Likert scale ranging from 0 (disagree) to 3 (agree). The total score ranged from 0 to 30, with a higher total score indicating more positive attitudes towards seeking professional psychological help. The reliability and efficacy of the Chinese version of ATSPPH-SF have been verified among community residents in China (10). In this study, Cronbach’s alpha coefficient for women with PND and their spouses were 0.855 and 0.871 on this scale, respectively.

#### 2.4.4 Stigma for seeking professional psychological help (SSPPH)

The Stigma Scale for Seeking Professional Psychological Help (SSPPH) was used to measure participants’ stigma associated with seeking professional psychological help (36). SSPPH consisted of a 5-item public stigma scale and a 5-item self-stigma scale. This study used a 5-point Likert scale rating from 1 (strongly disagree) to 5 (strongly agree). The total score ranged from 10 to 50, with higher scores indicating more significant stigma. The Chinese version of SSPPH showed good reliability and validity in a Chinese sample (22). In this study, women with PND and their spouses had Cronbach’s alpha coefficient of 0.906 and 0.925 on this scale, respectively.

#### 2.4.5 General help-seeking questionnaire (GHSQ)

The General Help-Seeking Questionnaire (GHSQ) was developed by Wilson and Rickwood et al. in 2005 to assess an individual’s to intention to seek help from different sources to cope with emotional problems (37). GHSQ consisted of three dimensions, representing different intentions to seek help. This study used the Intention to Seek Professional Psychological Help (GHSQ-ISPH) dimension, which includes psychological helpline, psychological professionals, general practitioners or perinatal nurses, to assess the intention of PND women and their spouses to seek professional psychological help. This study used a 7-point Likert scale ranging from 1 (never) to 7 (very likely). The total score ranged from 3 to 21, with higher scores indicating a greater intention to seek help from these sources. The Chinese version of GHSQ demonstrated good internal consistency (Cronbach’s alpha coefficient was 0.85) (38). In this study, women with PND and their spouses had Cronbach’s alpha coefficient of 0.774 and 0.789 on this scale, respectively.

#### 2.4.6 Self-efficacy for seeking mental health care (SE-SMHC)

The Self-Efficacy for Seeking Mental Health Care (SE-SMHC) was developed by Moore et al. in 2015 to measure an individual’s belief in competence when seeking mental health care (39). SE-SMHC was a nine-item self-report scale consisting of knowledge competence beliefs and coping competence beliefs. This study used a 10-point Likert scale ranging from 1 (not confident) to 10 (very confident). The total score ranged from 9 to 90, with higher scores indicating higher self-efficacy. The Chinese version of SE-SMHC showed good reliability and validity in a Chinese sample (40). In this study, women with PND and their spouses had Cronbach’s alpha coefficient of 0.854 and 0.846 on this scale, respectively.

### 2.5 Ethical considerations

The study adhered to the ethical principles of the Declaration of Helsinki and received ethical approval from the institutional review board at the First Maternity and Infant Hospital Affiliated with Tongji University (KS21273). Before data collection, the researcher detailed the study’s purpose, methods, and significance to the eligible participants. Participants were also given the opportunity to ask any questions about the study before fully agreeing to participate. After asking for consent, all participants signed informed consent forms. All participants had the right to withdraw from the study at any time.

## 3 Statistical analyses

To ensure the accuracy of data entry, the data was initially entered into Excel by two researchers and then checked by a third researcher.

Latent profile analysis (LPA) is a person-centred analysis technique that divides individuals into groups of individuals who are similar to each other and different from other groups (41), and allows the proportions of the distribution of each group to be obtained so that the characteristics of each group can be explored further (42). By concentrating on individual heterogeneity, LPA aids in comprehending the traits and influencing factors of diverse potential profiles. Mplus 7.0 was used to establish the latent profile classification model. LPA was performed using the individual item scores of the GHSQ-ISPH as the explicit variables.

The optimal fit test of LPA was based on the following fit indices: (1) Akaike information criteria (AIC), Bayesian information criteria (BIC) and adjusted BIC (aBIC). The lower the AIC, BIC, aBIC values, the better the fit; (2) Entropy is used to evaluate the accuracy of the model classification and its value ranges from 0 to 1. When the Entropy is >0.8, the classification accuracy reaches 90%; (3) LoMendell-Rubin (LMR) and Bootstrapped Likelihood Ratio Test (BLRT), when the *p* < 0.05 corresponding to LMR and BLRT, it indicates that the class k model is superior to the class K-1 model. Starting from a single model, the number of categories was gradually increased, each fit index was comprehensively evaluated, and the optimal model was selected.

After determining the optimal potential profile model, SPSS 23.0 software was used to explore the influencing factors associated with different profiles. According to the type of data, Mean ± Standard deviation (SD) was used to describe quantitative and frequency distribution was used to describe qualitative data. Missing values were handled by direct deletion and filling in the missing values with Mean and Median. The Chi-Square test, Fisher’s exact test, Mann–Whitney U test and analysis of variance were used to compare sociological characteristics, GHSQ-ISPH, ATSPPH-SF, SE-SMHC and SSPPH among different potential categories. Multiple logistic regression was used to explore the factors influencing different potential categories of women with PND and their spouses’ intention to seek professional psychological help. All statistical tests were conducted two-tailed with a significance level of *α* = 0.05.

## 4 Results

### 4.1 Socio-demographic characteristics of women with PND and their spouses

Prior to the survey, we invited 332 couples to participate in the study. Of the couples, 18 wives and 30 husbands refused to participate. The proportion of wives who agreed to participate but whose husbands refused was 5.4%. Finally, 282 paper questionnaires were distributed to women with PND and their spouses. Taking into account data matching, 267 valid questionnaires were successfully collected from both women with PND and their spouses, achieving a recovery rate of 94.68%. Table 1 presents a comprehensive overview of the demographic and clinic characteristics.

**Table 1:** Comparison of socio-demographic characteristics between different profiles of women with PND and their spouses’ GHSQ-ISPH [*N* (%)].

Variables		Women with PND (*N* = 267)						Spouses (*N* = 267)					
		Total sample, *n* (%)	Profile 1 (*n* = 51)	Profile (*n* = 61)	Profile 3 (*n* = 155)	*X^2^*	*P*	Total sample, *n* (%)	Profile 1 (*n* = 51)	Profile (*n* = 61)	Profile 3 (*n* = 155)	*X^2^*	*P*
Ethnicity	Ethnic Han	259 (97.0)	50 (98.0)	58 (95.1)	151 (97.4)	1.118^b^	0.605	262 (98.1)	50 (98.0)	60 (98.4)	152 (98.1)	0.317^b^	1.000
	Ethnic minority	8 (3.0)	1 (2.0)	3 (4.9)	4 (2.6)			5 (1.9)	1 (2.0)	1 (1.6)	3 (1.9)		
Residence	Urban	135 (50.6)	26 (51.0)	32 (52.5)	77 (49.7)	0.140^a^	0.956	152 (56.9)	29 (56.9)	30 (49.2)	93 (60.0)	2.090^a^	0.351
	Rural	132 (49.4)	25 (49.0)	29 (47.5)	78 (50.3)			115 (43.1)	22 (43.1)	31 (50.8)	62 (40.0)		
Age	18–25 years	12 (4.5)	2 (3.9)	4 (6.6)	6 (3.9)	4.738^b^	0.786	4 (1.5)	1 (2.0)	0 (0)	3 (1.9)	3.405^b^	0.922
	26–30 years	89 (33.3)	21 (41.2)	21 (34.4)	47 (30.3)			73 (27.3)	15 (29.4)	15 (24.6)	43 (27.7)		
	31–35 years	128 (47.9)	20 (39.2)	27 (44.3)	81 (52.3)			126 (47.2)	23 (45.1)	33 (54.1)	70 (45.2)		
	36–40 years	34 (12.7)	7 (13.7)	8 (13.1)	19 (12.3)			52 (19.5)	11 (21.6)	11 (18.0)	30 (19.4)		
	>40 years	4 (1.5)	1 (2.0)	1 (1.6)	2 (1.3)			12 (4.5)	1 (2.0)	2 (3.3)	9 (5.8)		
Education	Postgraduate and above	46 (17.2)	12 (23.5)	13 (21.3)	21 (13.5)	15.719^a^	0.30^*^	47 (17.6)	14 (27.5)	8 (13.1)	25 (16.1)	10.832^a^	0.092
	University or college	176 (65.9)	25 (49.0)	37 (60.7)	114 (73.5)			177 (66.3)	28 (54.9)	39 (63.9)	110 (71.0)		
	High school or junior college	23 (8.6)	6 (11.8)	6 (9.8)	11 (7.1)			29 (10.9)	7 (13.7)	11 (18.0)	11 (7.1)		
	Junior high school	21 (7.9)	8 (15.7)	4 (6.6)	9 (5.8)			14 (5.2)	2 (3.9)	3 (5.0)	9 (5.8)		
	Primary and below	1 (0.4)	0 (0)	1 (1.6)	0 (0)			0 (0)	0 (0)	0 (0)	0 (0)		
Employment status	Full-time job	232 (86.9)	45 (88.2)	55 (90.2)	132 (85.2)	7.495^b^	0.299	258 (96.6)	50 (98.0)	61 (100.0)	147 (94.8)	3.386^b^	0.117
	Part-time job	4 (1.5)	0 (0)	0 (0)	4 (2.6)			9 (3.4)	1 (2.0)	0 (0)	8 (5.2)		
	Unemployment	20 (7.5)	6 (11.8)	4 (6.6)	10 (6.5)			-	-	-	-		
	Students	9 (3.4)	0 (0)	1 (1.6)	8 (5.2)			-	-	-	-		
	No working	2 (0.7)	0 (0)	1 (1.6)	1 (0.6)			-	-	-	-		
Previous medical experience	Unsatisfied	13 (4.9)	4 (7.8)	4 (6.6)	5 (3.2)	2.623^b^	0.272	14 (5.2)	3 (5.9)	5 (8.2)	6 (3.9)	1.944^b^	0.412
	Satisfied	254 (95.1)	47 (92.2)	57 (93.4)	150 (96.8)			253 (94.8)	48 (94.1)	56 (91.8)	149 (96.1)		
Marital relationship	Very good	229 (85.8)	44 (86.3)	50 (82.0)	135 (87.1)	2.398^b^	0.651	229 (85.8)	44 (86.3)	50 (82.0)	135 (87.1)	2.398^b^	0.651
	Good	34 (12.7)	7 (13.7)	9 (14.8)	18 (11.6)			34 (12.7)	7 (13.7)	9 (14.8)	18 (11.6)		
	Average	4 (1.5)	0 (0)	2 (3.3)	2 (1.3)			4 (1.5)	0 (0)	2 (3.3)	2 (1.3)		
History of mental illness	Yes	7 (2.6)	4 (7.8)	1 (1.6)	2 (1.3)	5.320^a^	0.047^*^	5 (1.9)	2 (3.9)	0 (0)	3 (1.9)	2.101^b^	0.298
	No	260 (97.4)	47 (92.2)	60 (98.4)	153 (98.7)			262 (98.1)	49 (96.1)	61 (100.0)	152 (98.1)		
History of seeking psychological help	Yes	13 (4.9)	1 (2.0)	1 (1.6)	11 (7.1)	3.230^b^	0.212	11 (4.1)	3 (5.9)	0 (0)	8 (5.2)	3.779^b^	0.145
	No	254 (95.1)	50 (98.0)	60 (98.4)	144 (92.9)			256 (95.9)	48 (94.1)	61 (100.0)	147 (94.8)		
Knowledge of PND	Yes	139 (52.1)	21 (41.2)	27 (44.3)	91 (58.7)	6.653^b^	0.036^*^	105 (39.3)	23 (45.1)	17 (27.9)	65 (41.9)	4.510^a^	0.104
	No	128 (47.9)	30 (58.8)	34 (55.7)	64 (41.3)			162 (60.7)	28 (54.9)	44 (72.1)	90 (58.1)		
Do you agree “sitting the month”?	Agree	257 (96.3)	49 (96.1)	58 (95.1)	150 (96.8)	0.654^b^	0.824	245 (91.8)	46 (90.2)	58 (95.1)	141 (91.0)	1.184^a^	0.564
	Disagree	10 (3.7)	2 (3.9)	3 (4.9)	5 (3.2)			22 (8.2)	5 (9.8)	3 (4.9)	14 (9.0)		
Number of children	One	191 (71.5)	37 (72.5)	41 (67.2)	113 (72.9)	3.203^b^	0.517	192 (71.9)	37 (72.5)	42 (68.9)	113 (72.9)	4.311^b^	0.336
	Two	68 (25.5)	13 (25.5)	16 (26.2)	39 (25.2)			68 (25.5)	14 (27.5)	15 (24.6)	39 (25.2)		
	Three or more	8 (3.0)	1 (2.0)	4 (6.6)	3 (1.9)			7 (2.6)	0 (0)	4 (6.6)	3 (1.9)		
Monthly income (USD)	>1714	99 (37.1)	19 (37.3)	18 (29.5)	62 (40.0)	3.283^a^	0.919	154 (57.7)	30 (58.8)	35 (57.4)	89 (57.4)	12.856^a^	0.037^*^
	1,286–1714	54 (20.2)	12 (23.5)	12 (19.7)	30 (19.4)			61 (22.8)	12 (23.5)	7 (11.5)	42 (27.1)		
	858–1,285	55 (20.6)	9 (17.6)	16 (26.2)	30 (19.4)			42 (15.7)	9 (17.6)	15 (24.6)	18 (11.6)		
	429–857	33 (12.4)	6 (11.8)	9 (14.8)	18 (11.6)			10 (3.7)	0 (0)	4 (6.6)	6 (3.9)		
	<429	26 (9.7)	5 (9.8)	6 (9.8)	15 (9.7)			-	-	-	-		
Stage of pregnancy	before delivery	132 (49.4)	26 (51.0)	31 (50.8)	75 (48.4)	0.164^a^	0.921						
	after delivery-one year after delivery	135 (50.6)	25 (49.0)	30 (49.2)	80 (51.6)								
Level of PND	Mild depression	203 (76.0)	41 (80.4)	44 (72.1)	118 (76.1)	1.042^a^	0.594						
	Severe depression	64 (24.0)	10 (19.6)	17 (27.9)	37 (23.9)								

*P < 0.05.

a, Pearson’s chi-squared test.

b, Fisher’s precision probability test.

Sitting the month: the phrases “sitting the month” refer to a traditional practice among Chinese women that encompasses postpartum health care. This practice typically involves a month-long period during which new mothers remain at home to rest and recuperate following childbirth.

### 4.2 Intention of women with PND and their spouses to seek professional psychological help

The mean scores of each item in GHSQ-ISPH were shown in Table 2. The mean scores of women with PND and their spouses in the GHSQ-ISPH, “psychological helpline,” “psychological professionals,” “general practitioner or perinatal nurses” were (12.17 ± 4.06) and (12.61 ± 3.88), (3.29 ± 1.63) and (3.48 ± 1.62), (4.54 ± 1.68) and (4.67 ± 1.49), (4.34 ± 1.59) and (4.46 ± 1.53), respectively. Spouses scored higher on both the GHSQ-ISPH and each item than women with PND, but the difference was not statistically significant (*p* = 0.199).

**Table 2:** The mean scores of each item in GHSQ-ISPH of women with PND and spouses (Mean ± SD).

Items	Women with PND (*N* = 267)	Spouses (N = 267)	*t*	*P*
**GHSQ-ISPH**	12.17 ± 4.06	12.61 ± 3.88	−1.286	0.199
Psychological helpline	3.29 ± 1.63	3.48 ± 1.61	−1.365	0.173
Psychological professionals	4.54 ± 1.68	4.67 ± 1.49	−1.008	0.314
General practitioners or perinatal nurses	4.34 ± 1.59	4.46 ± 1.53	−0.833	0.405

t, independent sample *t*-test. GHSQ, The General Help-Seeking Questionnaire-the Intention to Seek Professional Psychological Help.

### 4.3 Correlation analysis of PND women and their spouses’ GHSQ-ISPH

The results showed that ATSPPH-SF and SE-SMHC were positively related to GHSQ-ISPH (*p* < 0.01), and SSPPH was negatively related to GHSQ-ISPH (*p* < 0.01) in women with PND. ATSPPH-SF and SE-SMHC were positively related to GHSQ-ISPH (*p* < 0.01), and SSPPH (excluding self-stigma) were negatively correlated to GHSQ-ISPH (*p* < 0.05 or *p* < 0.01) in spouses. See Table 3.

**Table 3:** Correlations among ATSPPH-SF, SE-SMHC and SSPPH and GHSQ-ISPH in women with PND and their spouses.

Items	GHSQ-ISPH (Women with PND, *N* = 267)		GHSQ-ISPH (Spouses, *N* = 267)	
	*r*	*P*	*r*	*P*
**ATSPPH-SF**	0.448**	< 0.01	0.458**	< 0.01
Openness to seeking treatment for emotional problems	0.316**	< 0.01	0.292**	< 0.01
Value and need in seeking treatment	0.432**	< 0.01	0.484**	< 0.01
**SE-SMHC**	0.308**	< 0.01	0.347**	< 0.01
Knowledge competence beliefs	0.331**	< 0.01	0.345**	< 0.01
Coping competence beliefs	0.229**	< 0.01	0.294**	< 0.01
**SSPPH**	−0.331**	< 0.01	−0.157*	< 0.05
Public stigma	−0.361**	< 0.01	−0.197**	< 0.01
Self stigma	−0.216**	< 0.01	−0.086	0.162

*P < 0.05; **P < 0.01. ATSPPH-SF, Attitudes toward seeking professional psychological help scale–short form; SE-SMHC, Self-efficacy for seeking mental health care; SSPPH, Stigma for seeking professional psychological help.

### 4.4 Potential categories of women with PND and their spouses’ GHSQ-ISPH

Based on the LPA analysis of each item of GHSQ-ISPH, 0 ~ 4 potential profile models were built in turn. As the number of categories increased, the AIC, BIC and aBIC gradually decreased until the LMRT index of model 4 was not statistically significant (*p* > 0.05). The entropy of different categories was all >0.8, with model 3 having the highest entropy, indicating the highest classification accuracy. After a comprehensive comparison of fit indices selected, model 3 as the best-fitting model (Table 4).

**Table 4:** Model fit indices for latent profile analysis.

Models	AIC	BIC	aBIC	LMR (*P*)	BLRT (*P*)	Entropy	Profile [n(%)]			
							Profile 1	Profile 2	Profile 3	Profile 4
Model 1	6041.4	6084.5	6046.4	-	-	-	267			
Model 2	5,787	5855.2	5794.9	<0.001	<0.001	0.826	92 (34.46%)	175 (65.54%)		
Model 3	**5669.5**	**5762.7**	**5680.3**	**0.003**	**<0.001**	**0.852**	**51 (19.10%)**	**61 (22.85%)**	**155 (58.05%)**	
Model 4	5606.3	5724.7	5620.0	0.272	<0.002	0.802	55 (20.60%)	49 (18.35%)	106 (39.70%)	57 (21.35%)

AIC, Akaike information criteria; BIC, Bayesian information criteria; aBIC, adjusted BIC; LMR, LoMendell-Rubin; BLRT, Bootstrapped Likelihood Ratio Test. The bold part indicates the best-fitting model.

**Figure 1:**
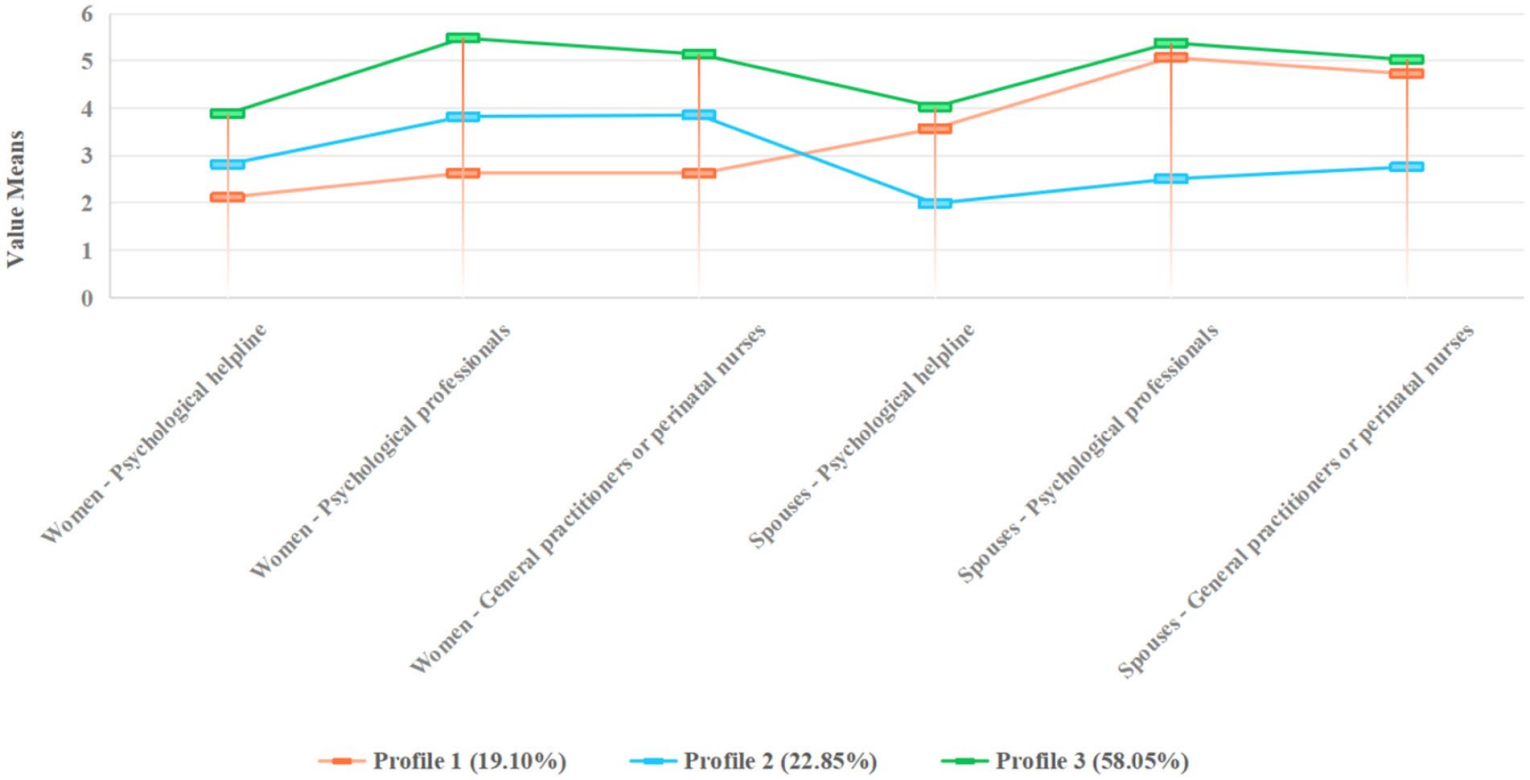
Latent profile models of the intention to seek professional psychological help among women with PND and their spouses.

The profile descriptions were based on comparisons between “psychological helpline,” “psychological professionals,” and “general practitioners or perinatal nurses.” These indicators were presented as mean values and standard deviations (Figure 1). Of the three intentions to seek professional psychological help latent categories, the scores for all items were higher for both women with PND in Profile 3 and their spouses, whereas the opposite was true for women in Profile 1 or Profile 3 and their spouses. The profiles were named according to the degree of the intention to seek professional psychological help, with Profile 1 (19.10%), Profile 2 (22.85%) and Profile 3 (58.05%) being named “Women low-Spouses high intention for help,” “Women high-Spouses low intention for help” and “Couple high intention for help” respectively.

### 4.5 Univariate analysis of potential profile categories influences on GHSQ-ISPH

The socio-demographic characteristics of women and their spouses with PND in the three potential categories were shown in Table 1. The results showed that history of mental illness, knowledge of PND, and education were statistically significant correlated to the GHSQ-ISPH (all *p* < 0.05) in women with PND, and monthly income.

ATSPPH-SF, SE-SMHC, and SSPPH (excluding self-stigma) were statistically significantly correlated to the GHSQ-ISPH (*p* < 0.01 or *p* < 0.05) in women with PND. ATSPPH-SF, SE-SMHC and public stigma of spouses were statistically significant in all three potential categories (*p* < 0.01 or *p* < 0.05), as shown in Table 5.

**Table 5:** Comparison of ATSPPH-SF, SE-SMHC, SSPPH scores for 3 potential profile categories (Mean, SD).

	Women with PND (*N* = 267)						Spouses (*N* = 267)					
	Profile 1 (*n* = 51)	Profile 2 (*n* = 61)	Profile 3 (*n* = 155)	Profiles-multiple comparison	*F*	*p*	Profile 1 (*n* = 51)	Profile 2 (*n* = 61)	Profile 3 (*n* = 155)	Profiles-multiple comparison	*F*	*p*
**ATSPPH-SF**	16.06 (2.831)	16.54 (3.418)	17.95 (2.679)^①③^	1<2<3	10.677	<0.001	17.76 (2.937)	14.54 (3.042)^③^	17.59 (2.949)^①③^	2<3<1	25.573	<0.001
Openness to seeking treatment for emotional problems	7.35 (1.730)	7.67 (2.150)	8.25 (1.952)^①^	1<2<3	4.737	0.010	8.06 (2.034)	6.66 (1.852)^③^	7.97 (1.934)^①③^	2<3<1	11.312	<0.001
Value and need in seeking treatment	8.71 (1.432)	8.87 (1.803)	9.70 (1.559)^①③^	1<2<3	10.690	<0.001	9.71 (1.474)	7.89 (1.603)^③^	9.61 (1.800)^①③^	2<3<1	25.059	<0.001
**SE-SMHC**	57.90 (12.708)	56.07 (16.111)	64.83 (14.949)^①③^	2<1<3	9.494	<0.001	61.53 (12.511)	53.74 (16.018)^③^	64.15 (14.577)^③^	2<1<3	11.218	<0.001
Knowledge competence beliefs	30.51 (8.118)	29.61 (9.107)	35.21 (9.432)^①③^	2<1<3	10.652	<0.001	33.43 (8.293)	28.20 (9.159)^③^	35.14 (8.935)^③^	2<1<3	13.437	<0.001
Coping competence beliefs	27.39 (6.178)	26.46 (7.667)	29.62 (6.606)^②③^	2<1<3	5.546	0.004	28.10 (6.152)	25.54 (8.034)^②^	29.01 (6.486)^③^	2<1<3	5.691	0.004
**SSPPH**	23.06 (4.393)	22.11 (5.547)	20.25 (5.378)^①④^	3<2<1	6.696	0.001	23.24 (5.260)	23.51 (5.673)	22.01 (5.667)	3<1<2	2.005	0.137
Public stigma	12.10 (2.773)	11.13 (3.319)	9.94 (2.975)^①③^	3<2<1	10.959	<0.001	11.27 (2.793)	11.72 (3.158)	10.37 (3.077)^③^	3<1<2	4.940	0.008
Self stigma	10.96 (2.425)	10.98 (3.122)	10.31 (3.078)	3<1<2	1.617	0.200	11.96 (3.168)	11.79 (3.205)	11.64 (3.146)	3<2<1	0.210	0.811

①: comparison with profile 1, *P* < 0.01; ②: comparison with profile 1, *P* < 0.05; ③: comparison with profile 2, *P* < 0.01; ④: comparison with profile profile 2, *P* < 0.05. ATSPPH-SF, Attitudes toward seeking professional psychological help scale–short form; SE-SMHC, Self-efficacy for seeking mental health care; SSPPH, Stigma for seeking professional psychological help.

### 4.6 Factors influencing women with PND and their spouses’ GHSQ-ISPH

The potential categories of women with PND and their spouses’ GHSQ-ISPH were used as the dependent variable, and Profile3 was used as the reference group. Statistically significant factors in the univariate analysis were used as independent variables in the multiple logistic regression analysis. The statistically significant results of multiple logistic regression were shown in Table 6, with Profile 3 treated as the reference group. Women’ public stigma, women’ education, spouses’ knowledge competence beliefs, spouses’ ATSPPH-SF, spouses’ openness to seeking treatment for emotional problems and spouses’ monthly income were significantly associated with these three profiles (*p* < 0.05 or *p* < 0.01).

**Table 6:** Multifactor analysis of women with PND and their spouses’ GHSQ-ISPH by multiple logistic regression.

	*Β*	S.E.	Wald *χ*^2^	*P*	OR	95% CI
Profile 1 vs Profile 3 (ref)						
Women’ public stigma	0.382	0.146	6.824	0.009**	1.465	1.100,1.950
Women’ education (university or college)	−1.184	0.511	5.371	0.020*	0.306	0.112,0.833
Profile 2 vs Profile 3 (ref)						
Spouses’ knowledge competence beliefs	−0.066	0.031	4.372	0.037*	0.937	0.881,0.996
Spouses’ monthly income (858–1,285 USD)	1.102	0.533	4.269	0.039*	3.009	1.058,8.556
Spouses’ ATSPPH-SF	−0.577	0.136	18.014	<0.001	0.561	0.430,0.733
Spouses’ openness to seeking treatment for emotional problems	0.454	0.207	4.813	0.028*	1.575	1.050,2.364

ref, reference; S.E., stander error; OR, odds ratio; 95% C.I, confidence Interval; *P < 0.05; ** *P* < 0.01.

Specifically, spouses who monthly income was 858–1,285 USD were more likely to be members of Profile 2 than Profile 3 (OR = 3.009, 95%CI = 1.058–8.556, *p* = 0.039), and women who received a university or college education were more likely to be members of Profile 3 than Profile 1 (OR = 0.306, 95%CI = 0.112–0.833, *p* = 0.020). With respect to public stigma, a higher score was significantly associated with women with PND with Profile 1 than profile 3 (OR = 1.465, 95%CI = 1.100–1.950, *p* = 0.009). A lower openness to seeking treatment for emotional problems was significantly associated with spouses with Profile 2 than Profile 3 (OR = 1.575, 95%CI =1.050–2.364, *p* = 0.028). Compared with the profile 2, spouses in Profile 3 had higher knowledge competence beliefs scores (OR = 0.937; 95%CI = 0.881–0.996, *p* = 0.037) and women who received a university or college education were more likely (OR = 0.561, 95%CI = 0.430–0.733, *P* < 0.001), as seen in Table 6.

## 5 Discussion

### 5.1 Latent profile of couples’ GHSQ-ISPH and demand for professional psychological help during the perinatal period

Through latent profile analysis, this study identified three distinct profiles of couples based on their GHSQ-ISPH scores: “Women low-Spouses high intention for help (Profile 1),” “Women high-Spouses low intention for help (Profile 2)” and “Couple high intention for help (Profile 3).” These findings revealed heterogeneity in the intention of women with PND and their spouses to seek professional psychological help. Among these profiles, Profile 3 accounted for only 58.05%. The results of this study showed that the mean GHSQ-ISPH scores of women with and their spouses were 14.50 ± 2.664 and 14.46 ± 2.697 respectively, reflecting their intention to seek professional psychological help at a moderate level, lower than the research results of a previous study (19).

The scores of all entries from women with PND and their spouses in Profile 1 or Profile 2 were significantly lower than Profile 3. One possible explanation for this, couples are more likely to seek help from their partners or community health services (19, 43). Shumet et al. (43) found that people tend to choose community health services close to home when they perceive their health condition to be relatively minor. Acknowledging the people’s inclination towards community health services, perinatal nurses are accordingly encouraged to work in conjunction with these services to bolster maternal and child health initiatives. This collaborative effort is intended to facilitate access to formal assistance for couples grappling with serious problems and in need of professional psychological support.

In Profile 3, both women with PND and their spouses predominantly sought assistance from psychological professionals, with general practitioners or perinatal nurses being the secondary choice. During the perinatal period, nurses, known for their close contact with couples, were surprisingly not reported as the primary resource in this study. As identified by Prevatt et al. (44) the pivotal factor in accessing treatment, regardless of screening, was women’s willingness to disclose their emotional problems. Furthermore, the proactive approach of healthcare providers in offering mental health services and encouraging women’s engagement significantly facilitated women’s disclosure of symptoms. In line with the guidelines of the American College of Obstetricians and Gynaecologists’s guidelines (45), perinatal nurses are advised to conduct at least one comprehensive assessment of a woman’s emotional health during the perinatal period. In contrast, the 2018 guidelines for preconception and prenatal care in China (46) only broadly emphasize the importance of addressing women’s mental health, without specifying detailed recommendations to improve the detection and management of PND, it is recommended that women undergo at least one screening for PND. Updating China’s guidelines to include explicit recommendations on the timing and frequency of mental health assessments for obstetric providers is essential to ensure more effective and standardized care.

### 5.2 The influence of socio-demographic characteristics on women with PND and their spouses’ GHSQ-ISPH

Our study found that education, knowledge of PND and history of mental illness for women with PND and spouses’ monthly income were factors influencing couples’ GHSQ-ISPH through univariate and multiple logistic regression analyses. Women with PND in Profile 1, who often have little knowledge of PND. Insufficient awareness of PND may lead to women being unaware of PND symptoms, thereby hindering them from seeking psychological help (47). The cognition of PND in this study was lower than the findings of Ayres et al. (48). Therefore, perinatal mental health education is essential to bridge the knowledge gap regarding PND among women. Currently, there is a lack of specialized training for perinatal nurses in China to ensure that women receive vital information about PND throughout the perinatal phase. To address this gap, comprehensive training programs in mental health services should be developed and integrated into the regular training of perinatal nurses working in both outpatient and inpatient settings. These programs should focus on equipping nurses with the skills necessary to provide high-quality antenatal and postnatal care, including standardized nursing processes. In recent efforts to improve training, some hospitals in China have introduced systematic training programs and smart app-based courses. These app-based courses enhance learning by offering flexibility, interactive modules, and situational reproducibility (49, 50). History of mental illness was an influencing factor of GHSQ-ISPH in women with PND. Women with PND without a history of mental illness were more likely to be classified as Profile 3. Consistent with the findings of Nagle et al. (51), PND women who reported a history of mental illness demonstrated awareness and insight into their own mental health, conveying confidence in their ability to manage mental problems.

The multiple logistic regression analyses found that education was the influencing factor of PND women’s CHSQ-ISPH, consistent with the research of Huang et al. (22). Education serves as the cornerstone for numerous aspects of society, enabling individuals with higher educational attainment to better understand and effectively engage with professional psychological services (30). The lack of awareness of PND, coupled with inadequate mental health support from obstetricians and nurses, discourages women from seeking help, even in resource-rich cities like Shanghai (27, 52). To address this, tailored recommendations have been proposed to improve access to psychological knowledge and services. First, outpatient and resident nurses should provide health education manuals covering prenatal and postnatal care, including mental health information. Second, obstetricians and nurses must proactively educate women on perinatal psychological issues, risk factors, and symptoms during prenatal visits, offering counseling tailored to their educational background (53). Women with severe psychological problems should be referred to specialized institutions. Lastly, for women undergoing psychotherapy, obstetricians should ensure follow-up care, ideally in collaboration with psychological institutions and community health services in resource-abundant areas. These measures aim to bridge educational disparities and meet women’s cognitive and mental health needs effectively.

In this study, monthly income was the influencing factor of spouses’ GHSQ-ISPH. Spouses with higher incomes were more likely to be grouped into Profile 2 or Profile 3. Consistent with the research of Luís et al. (21), higher-income spouses were more likely to use health services and refer their wives to professionals for help, especially during the postpartum period. The low intention of low-income spouses to seek professional psychological help might be related to their inability to afford costs for psychological services or to their insufficient income to fulfil health insurance requirements. Currently, psychotherapy coverage in lower-middle-income countries is only 14%, far below the target of 42% (54). The shortage of psychotherapists drives up the cost of therapy. To address this, we must expand access to psychological services. Future health policies should focus on increasing investment in mental health and enhancing health insurance coverage for better overall benefits.

### 5.3 The influence of attitudes toward seeking professional psychological help, self-efficacy and stigma on couples’ GHSQ-ISPH

This study showed that women with PND and their spouses with negative attitudes towards seeking professional psychological help were more likely to belong to Profile 1 or Profile 2, indicating that attitudes towards seeking professional psychological help are a factor for GHSQ-ISPH in couples. This aligns with the theory of Planned Behavior (TPB), which suggests that a positive attitude is a significant positive predictor of the intention to seeking professional help (55). Studies have shown that a more positive attitude towards professional psychological assistance is associated with a stronger inclination to seek such help, which in turn leads to an increase in the actual pursuit of professional help-seeking behavior (56). In this study, women with PND and their spouses who had a positive attitude towards seeking help scored higher on the “psychological professional” item. This finding is consistent with previous research that people with a lower willingness to seek professional psychological help are less likely to seek help from professionals (22). Jones et al. (57) also found that help-seeking attitudes were associated with mental health literacy, such as the ability to recognize mental health and the understanding and recognition of professional help. Perinatal nurses can explore PND treatments with couples to gain insight into their perspectives and beliefs about professional psychological support. Through this approach, early identification and intervention with couples who reluctant to seek professional psychological help may improve their perceptions and attitudes towards such help.

This study found that couples’ self-efficacy was associated with the GHSQ-ISPH. Women with PND and their spouses in Profile 1 or Profile 2 appeared to have low self-efficacy. The results were align with the findings of Garrey et al. (58), who found that people with high self-efficacy may feel more empowered to take care of their mental health problem independently, and as a result, may be less willing to seek help than people with low self-efficacy. Previous research had found that people with high self-efficacy have coping knowledge competence belief and higher coping knowledge competence (59). In this study, couple’ “knowledge competence beliefs” and “Coping competence beliefs” scores were positively associated with GHSQ-ISPH. A lack of awareness of PND and the psychological services available often prevents individuals, particularly couples with limited coping skills, from seeking help. This ultimately leads lacking the confidence in couples to seek professional psychological help. This finding is consistent with the findings of Tomczyk et al. (60) and can be explained by the TPB theory. Perinatal nurses can facilitate open dialog between couples about perinatal concerns, aiming to comprehend their past treatment experiences, existing knowledge, and preferences for support.

This study found that women with PND and their spouses who experienced higher stigma were more likely to be grouped into Profile 2 or Profile 1. We found that higher stigma was associated with negative attitudes towards seeking professional psychological help, which is consistent with previous findings (57, 61). This association can be attributed to the tendency of individuals with psychological problems to hide their problems and avoid treatment in order to mitigate the potential negative consequences of seeking mental health services (62). Notably, spouses in all three potential profile categories reported higher self-stigma compared to women with PND, which may reflect gender-cultural differences in male or female behavior when seeking professional psychological help (63). In our study, women in Profile 1 had the highest level of public stigma. In addition, in Chinese culture, mental problems are usually linked with signs of “sickness” and “incompetence,” as well as high expectations for women to be “good mothers,” leading to a reluctance to seek professionals when suffering from PND (22). The TPB theory considers behavioral attitudes, behavioral intentions, and subjective norms control as predictors of individual behavior (55). Our findings show that stigma is a barrier to couples’ intention to seek professional psychological help. This results in a negative subjective perception of PND and psychotherapy among couples, influencing their attitude towards psychological services, and indirectly causing a lack of behavior in seeking professional psychological assistance. Perinatal nurses play a crucial role in screening for stigmatized conditions. Due to their frequent interactions with couples, they are encouraged to create comfortable communication environments. Nurses should initiate conversations about parenting experiences and psychological concerns to identify couples who may be at a higher risk of facing stigma.

## 6 Limitations

The study has some limitations. Firstly, while the sample size adequately met statistical requirements, it was confined to participants sourced solely from a tertiary hospital in Shanghai. This sample may not be representative of all women with PND or their spouses, potentially introducing bias into the findings. Secondly, conducting surveys with women and their spouses together may lead to mutual interference in their responses. This could include biases in questionnaire answers and the potential influence of women’s PND on their spouses, either directly or indirectly. To mitigate issues of endogeneity and reverse causality, future research should consider studying women and their spouses independently to ensure more accurate and unbiased findings. Finally, this study was a cross-sectional study that only described the correlation between influencing factors and the intention to seek professional psychological help. It is important to note that intention does not necessarily translate into actual behavior, so the findings need further validation. Therefore, a multi-center, large-sample longitudinal study can be undertaken in the future to further substantiate the findings of this study.

## 7 Conclusion

The findings of this study indicate a moderate level of intention to seek professional psychological help among women experiencing PND and their partners. Several factors, including socio-demographic characteristics, attitudes towards help-seeking, stigma, and levels of self-efficacy, significantly influence this intention. Perinatal nurses should pay attention to women with PND and their spouses, especially those in Profile 1 and Profile 2, and adopt positive strategies to guide couples in Profile 3 to seek professional psychological help. The identified factors influencing help-seeking intentions underscore the importance of tailored interventions and strategies within perinatal mental health nursing. Such measures are essential not only for enhancing perinatal mental health education but also for informing relevant policy, ultimately aiming to increase the likelihood that couples will seek the help they need.

## Data Availability

The original contributions presented in the study are included in the article/supplementary material, further inquiries can be directed to the corresponding author/s.
